# Effects of Silica Aerogel Content on the Properties of Waterborne Organic Thermal Insulation Coatings

**DOI:** 10.3390/gels11070547

**Published:** 2025-07-15

**Authors:** Zikang Chen, Dingwei Li, Shengjie Yao, Yumin Duan, Jiahui Chen, Miao Liu, Taoying Liu, Zhi Li

**Affiliations:** School of Resource and Safety Engineering, Central South University, Changsha 410083, China; 235512117@csu.edu.cn (Z.C.); ldw237339@163.com (D.L.); 235511068@csu.edu.cn (S.Y.); 245511058@csu.edu.cn (Y.D.); 245511045@csu.edu.cn (J.C.); liumiao99@csu.edu.cn (M.L.)

**Keywords:** SiO_2_ aerogel, thermal insulation coatings, thermal insulation properties, comprehensive performance

## Abstract

In order to cope with the emergence of energy conservation and consumption reduction initiatives, we used an acrylic emulsion (as the adhesive), combined with silica aerogel (SA) and hollow glass microsphere (HGM) fillers, to synthesize thermal insulation coatings, which were found to have low thermal conductivity and excellent thermal insulation properties. These waterborne coatings are environmentally friendly and were synthesized without organic solvents. Comprehensive testing verified that the coatings met practical requirements. Specifically, the addition of 18% SA resulted in minimal thermal conductivity (0.0433 W/m·K), the lowest density (0.177 g/cm^3^), as well as a reduced gross calorific value. At a heating surface temperature of 200 °C, the 5 mm coating’s cooling surface temperature was 108.7 °C, yielding a 91.3 °C temperature difference and demonstrating remarkable thermal insulation performance. Furthermore, the coatings showed favorable results in terms of water resistance, corrosion resistance, wear resistance, and adhesion, achieving satisfactory engineering standards. In this work, the influence of different contents of SA on various properties of the coating was studied, with the aim of providing a reference for the modulation of the comprehensive performance of SA thermal insulation coatings.

## 1. Introduction

Against the backdrop of the global energy transition and the advancement of the “dual carbon” strategy, energy consumption has become an increasingly significant concern, leading to a growing demand for thermal insulation technologies to address this challenge [1,2]. Research on thermal insulation coatings will help promote the development of energy conservation and emission reduction technologies, facilitate the sustainable development of human society, and is also a key path for implement green and low-carbon development strategies [3,4,5,6,7]. Silica aerogel (SA) has garnered significant attention as a nano−porous material due to its extremely low density and superior thermal insulation properties. It is a porous material characterized by an exceptionally high specific surface area and micro-nano porosity. It is synthesized through chemical reactions involving amorphous silica, with air occupying its internal pore structure. Its unique three−dimensional network structure has brought about a revolutionary breakthrough in thermal insulation. Its thermal insulation mechanism is achieved through the synergy of three paths: On the solid-phase heat conduction path, nano-pores form nearly infinitely long phonon transfer channels, significantly prolonging the thermal resistance path. On the gas−phase thermal convection path, the micro−nano pore size limits the average free path of gas molecules, generating a “zero convection” effect. On the infrared thermal radiation path, the high density nano−pore walls form multiple scattering interfaces, effectively blocking radiative heat transfer [8]. Hence, the application of SA in the form of coatings in engineering not only greatly enhances its adaptability to different shapes, but also offers better construction convenience and adhesion to substrates to achieve the synergy of thermal insulation and durability. Compared with organic solvent-based coatings, waterborne thermal insulation coatings are more environmentally friendly and beneficial to human health. In recent years, the research and application of coatings have placed an increasing focus on waterborne coatings [9,10,11].

Kiil developed a mathematical heat transfer model for SA thermal insulation coatings, verifying that SA fillers demonstrate better thermal insulation properties than hollow glass microspheres (HGMs) and polymeric hollow beads. The model indicated an inverse correlation between filler volume concentration and coating thermal conductivity [12]. In formulation optimization studies, Liu et al. systematically screened components to develop a composite coating with a total filler percentage of 25 wt.% (5 wt.% SA, 5 wt.% TiO_2_, 5 wt.% HGMs, and 10 wt.% far-infrared ceramic powder). This configuration reduced the substrate temperature to 44.4 °C, 14.8 °C below the uncoated controls, validating SA’s critical role in enhancing thermal insulation and heat resistance [13]. Di’s study investigated SA−HGM composite coatings and revealed that SA outperformed HGMs in thermal insulation performance. However, increasing the SA mass fraction reduced the coating’s adhesive strength due to SA’s porous structure, which hinders the formation of a dense matrix. The optimal performance was obtained at a 1:1 HGM/SA mass ratio, where a balanced modulation of thermal conductivity (0.05 W/m·K) and adhesive strength (1024 KPa) was achieved [10]. He et al. further demonstrated progressive thermal conductivity reduction with increasing SA content, reaching a minimum thermal conductivity of 0.057 W/m·K with a 6 g dosage. For the 4 mm thick coating, the 351 °C heated surface induced a temperature of merely 188 °C on the cooling side, a 163 °C differential, exceeding the SA-free coatings by 29 °C [14]. Han et al. prepared a waterborne coating by adding porous calcium silicate and SA, producing a coating with a thermal conductivity of 0.0854 W/m·K and an exceptional radiative cooling ability. In the daytime test, the temperature inside the box achieved a cooling temperature 13 °C lower than the 30 °C environment, and the theoretical cooling power was 96 W/m^2^ [15]. Lei et al. synthesized modified SA nanoparticles using the sol−gel method using phytic acid and ethyl trimethoxysilane, and then introduced them into a waterborne epoxy coating. This coating exhibited excellent corrosion resistance and thermal insulation; the results showed that the corrosion inhibition efficiency was 99.9% higher than that of the pure coating. In addition, the lowest thermal conductivity of the SA coating was 0.0963 W/m·K and temperature of the incubator was 9.1 °C lower than that of the pure coating in the thermal insulation tests [16]. In summary, SA composite coatings have been frequently demonstrated to have excellent thermal insulation performance, but the negative impact of SA on the comprehensive performance of coatings cannot be ignored, such as mechanical brittleness and weak interface bonding between SA and the adhesive [17,18]. In addition, the role of SA in enhancing the heat resistance and combustion performance of organic coatings at high temperatures has received limited attention. Furthermore, it is also regrettable that the thermal insulation performance of SA coating sacrifices other functional properties. Therefore, it is important to balance the multifaceted performance of SA-based thermal insulation coatings.

However, due to the high purity demand and high price of SA, it is not economically feasible to add a large amount of SA as a filler in coatings. In addition, when SA is used as the only thermal insulation filler in a coating, the strength development of the coating also faces challenges. Therefore, it more reasonable to adjust the mechanical and thermal properties of the aerogel thermal insulation coating by adding SA and other low cost thermal insulation fillers [19]. Rigid HGMs with closed air cavities have been widely reported used as thermal insulation and toughening fillers in coatings due to their low density, strong chemical inertness, anti-deformation capacity, and excellent thermal insulation ability [20]. Studies have shown that the use of HGMs and SA in coating composites leads to high strength parameters and low thermal conductivity. Chen et al. used HGMs as the main functional filler, anionic emulsifiers as the adhesive and stabilizers, and zinc phosphate as the modifier. The lowest thermal conductivity of 0.157 W/m·K was observed with the coating with 15 wt.% modified HGMs. Furthermore, when the addition amount was 15wt%, the corrosion current density was the lowest [21]. Xing et al. prepared a thermal insulation composite using epoxy resin as the matrix and HGMs as the filler; the flexural strength and the flexural modulus of the composite reached as high as 22.34 ± 2.75 MPa and 1.34 ± 0.03 GPa, respectively. The composite exhibited low thermal conductivity and excellent thermal insulation, primarily due to the efficient obstruction of heat conduction paths by HGMs [22]. Parisa Niazi et al. prepared polyester based composite coatings using HGMs and fumed silica. The results confirmed that when the coating contained 10 wt.% HGMs and 12 wt.% fumed silica, it exhibited the greatest thermal insulating performance (around 20% improvement compared to the pure polyester) [23].

In this study, we chose a water-based acrylic emulsion as the adhesive and strategically combined lightweight thermal HGM insulation fillers with hydrophobic SA. The thermal insulation coating was fabricated via a simple physical mixing combined with mechanical dispersion approach. This study investigated the impact of SA content on the coating’s comprehensive performance characteristics.

## 2. Results and Discussion

### 2.1. Microstructure

**Figure 1:**
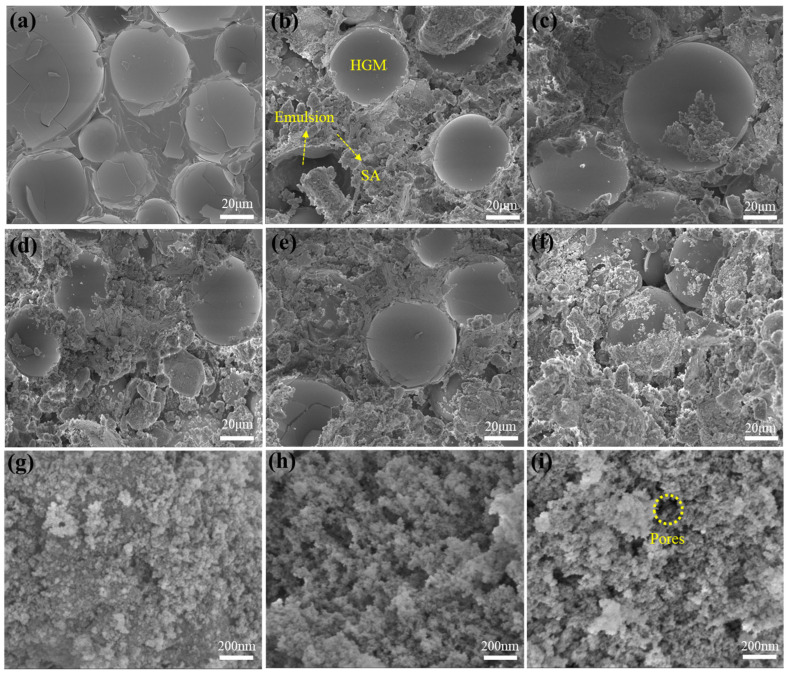
SEM images of coatings with different SA contents: (**a**) 0%, (**b**) 10%, (**c**) 12%, (**d**) 14%, (**e**) 16%, and (**f**) 18%. Micrographs of coating samples with different SA contents: (**g**) 10% (**h**) 14%, and (**i**) 18%.

Figure 1 illustrates the internal structure diagram of the coating. As depicted in Figure 1a, the HGMs present a “dispersed microsphere morphology” within the coating without the addition of SA, and were uniformly embedded in the polymer matrix without being tightly packed together. This is because HGMs are circular spheres filled with air, with a small specific surface area, lightweight properties, and good fluidity; thus, the interaction between the HGMs and the polymer matrix was strong [20]. There are clear polymer networks connecting the spheres. In this coating, the thermal insulation performance inside the coating comes from the HGMs. With the addition of nano−SA, the gaps between the HGMs gradually filled with more SA, which adheres to and grows on the surface of the HGMs. In the samples with 10% and 12% SA, the HGMs, with their characteristic spherical shape, were observed on the surface. When the SA content exceeded 12%, the HGMs gradually became embedded in the coating and were covered by the SA. The SA was densely connected to adjacent HGMs, forming a thermal conductivity barrier and reducing the solid-state heat transfer path. Figure 1g–i shows a magnified view of the bonding interface between the SA and the emulsion. It can be seen that the SA particles were cross−linked by the emulsion because the emulsion was unable to fully encapsulate the nanoporous pores. However, as the SA content increased, its porous structure weakened the compactness of the acrylic emulsion, and some pore channels appeared. This phenomenon resembles the structural characteristics of SA in cementitious materials, where composites containing SA display a loosely packed structure with numerous interconnected cracks. Additionally, distinct interface regions exist between the matrix and filler, which results in relatively weak bonding and highly porous regions known as interface transition zones [24].

### 2.2. Water Resistance 

**Figure 2:**
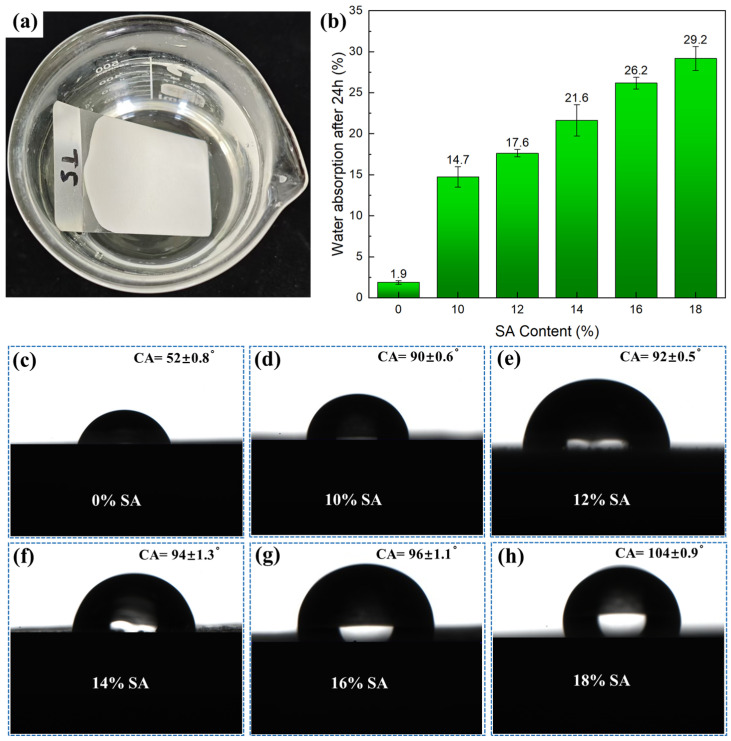
(**a**) Immersion of coating during water test; (**b**) water absorption rate of the coating after 24 h of soaking; (**c**–**h**) contact angle of surface of coatings with different SA contents.

The waterproof and water-resistant properties of the coating are important indicators for evaluating its reliability. Coatings with excellent water resistance can maintain structural integrity under long term humid environments or repeated water erosion, ensuring the stability of the adhesive and mechanical properties, and functional characteristics of the coating on the substrate. This directly reduces the full life−cycle cost. In this study, we coated two−thirds of a tinplate with a coating and immersed it in water for 24 h (Figure 2a). After taking out the samples, we absorbed the surface moisture with filter paper and visually inspected the test plates. All the samples showed no loss of gloss, discoloration, bubbling, wrinkling, or peeling, demonstrating excellent water resistance. Meanwhile, the weight gain of the test plate was recorded, and the water absorption rate is shown in Figure 2b. Although SA itself is hydrophobic, its nano-porous structure forms a penetrating pore network in the coating, which disrupts the weak interfacial bonds of the original continuous membrane structure in the acrylic emulsion. As shown in Figure 1g–i, a higher SA content more severely hindered the fusion of latex particles, transforming the coating from a continuous dense film into a porous structure and significantly increasing water absorption. Consequently, an increased SA content increased the water absorption rate. Compared with the SA-free coating, the water absorption rate of the coating with the highest SA content (18%) was 29.2%, an increase of 27.32%. Figure 2c−h shows the contact angle (CA) measurements, which showed the opposite trend. It can be seen that the SA-free coating surface exhibited hydrophilicity, with a CA of only 52 ± 0.8°, which was attributed to the hydrophilic carboxyl residues from the polymerization of the acrylic emulsion [25]. With the addition of hydrophobic SA, the CA of the coating surface gradually increased due to the hydrophobic methyl groups on the surface of the SA, and the coating became hydrophobic. When the content of SA was 18%, the CA reached 104 ± 0.9°. From these results, it can be seen that SA helps reduce the instantaneous wettability of the coating surface but increases the water absorption. This divergence arises from the different evaluation dimensions of the two properties: CA measures the “initial spreading tendency” of a liquid on the surface, while water absorption reflects the total amount of water absorbed by the entire coating over a specific period of time. This is not only related to surface wettability but also closely associated with the pore structure, connectivity, and capillary action of the composite coating [26]. Some studies have mentioned that the increased water absorption of SA composites in humid environments is related to the increase in pore connectivity and the poor interfacial adhesion and formation of microcracks between SA and the matrix [27,28]. This is worth studying in detail from a micro level in future work. However, from the existing engineering standards for testing the water absorption rate of coatings, such as HG/T 3344-2012 “Determination of water absorption of paint film” (China, 2012), the immersion method is more applicable [29].

### 2.3. Corrosion Resistance 

**Figure 3:**
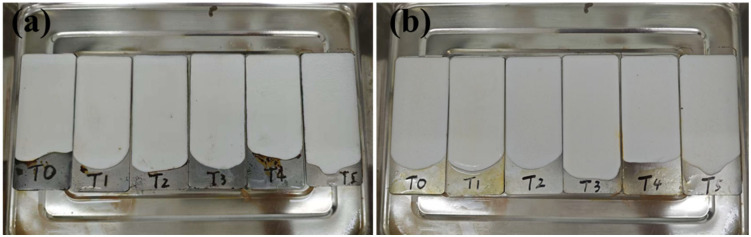
After immersion in (**a**) 5 wt.% NaOH or (**b**) 3.5 wt.% NaCl for 7 days.

Metallic corrosion represents a pervasive phenomenon with significant economic and safety implications, and is fundamentally driven by electrochemical mechanisms. When corrosive media (acids, alkalis, and salts) interact with protective coatings, they induce material degradation through solvation processes or by initiating micro-crazing. These structural compromises enable corrosive species to infiltrate the coating via inherent pores, voids, and diffusion pathways, ultimately compromising both the coating’s long-term integrity and thermal insulation performance [30]. In this study, we applied the coatings to tinplate sheets, soaked them in a 3.5 wt.% NaCl solution or 5 wt.% NaOH solution, and removed them after 7 days. The coatings remained intact without peeling, powdering, or cracking (Figure 3). SA and HGMs are chemically inert and resistant to corrosion by chemical media such as alkali and salts, effectively blocking the penetration of corrosive factors. In fact, coatings containing SA that can resist corrosion have been reported many times. For example, Shen et al. used epoxy resin as the adhesive and combined it with modified SA and carbon nanotubes to prepare a coating with excellent corrosion resistance and thermal insulation. This design yielded a stable micro-nano layered architecture, where SA−generated micropores within the coating created a dual barrier mechanism: physically obstructing corrosive media penetration while suppressing electrochemical electron transfer, thereby enhancing the corrosion protection. The coating achieved a thermal conductivity of 0.093 W/m·K, representing a 72% reduction compared to the pure epoxy coating. In thermal validation tests, the coated enclosures maintained a 12 °C lower surface temperature than the uncoated controls, demonstrating its practical thermal insulation efficacy [31]. In addition, waterborne acrylic is also often reported to have corrosion resistance and mechanical durability [32]. Notably, rust formed on uncoated substrate areas due to electrochemical corrosion of the metal, highlighting the critical role of the coating. Thus, the prepared SA composite coatings exhibited excellent corrosion resistance, which can extend the substrate’s service life.

### 2.4. Mechanical Properties

**Figure 4:**
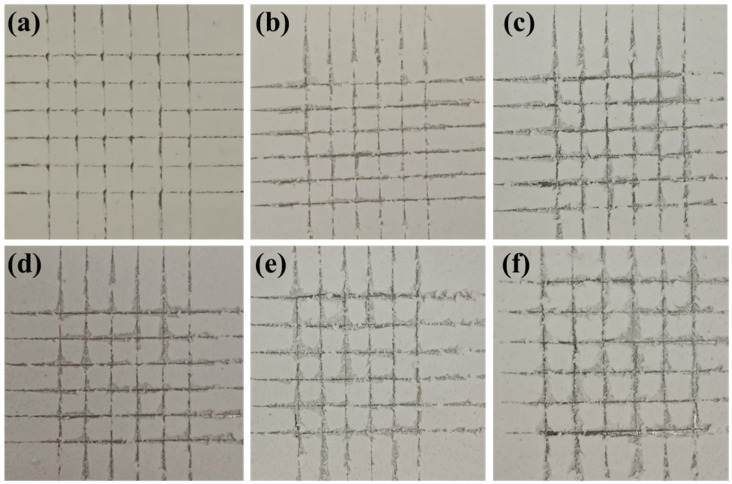
The adhesion grades of coatings with different SA contents evaluated using the cross−cut grid method: (**a**) 0%, (**b**) 10%, (**c**) 12%, (**d**) 14%, (**e**) 16%, and (**f**) 18%.

**Figure 5:**
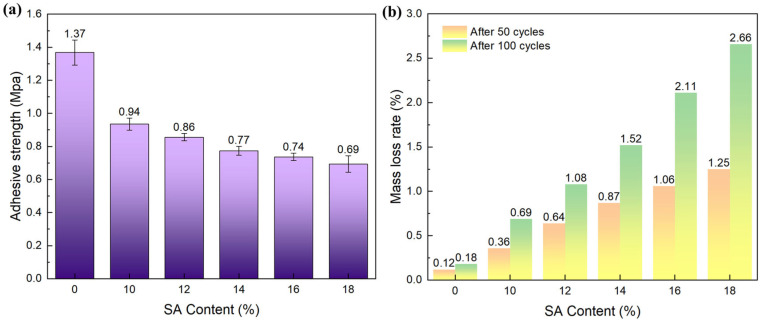
(**a**) The adhesive strength of the coatings was tested using the pull−out method. (**b**) Mass loss rate of coatings after 50 and 100 surface grinding cycles.

The mechanical integrity of thermal insulation coatings is a fundamental determinant of their sustained operational viability. Superior mechanical characteristics enable coatings to resist service induced stresses, including abrasion and impacts, while preserving structural continuity through crack suppression and delamination prevention. This investigation specifically evaluated the critical performance metrics of wear resistance and interfacial adhesion strength since durable bonding effectively mitigates thermal bridging risks arising from localized coating thinning and compromised insulation [33]. In this experiment, the cross−cut grid method was used to visually perceive the adhesion of the coating on the substrate, and the pull−out method was adopted to quantitatively describe the bonding strength between the coating and the substrate. Figure 4 shows that as the SA content increased over 14%, the amount of coating peeling gradually increased at the intersection of the cut, indicating decreasing coating adhesion. Figure 5a presents the quantitatively measured adhesive strength. It can be observed that the coating with 18% SA exhibited the lowest adhesive strength of 0.69 MPa, a 49.4% decrease compared to the SA-free coating. This can be attributed to two main factors: First, the loose and porous structure of SA introduces numerous voids into the coating’s internal structure, reducing its cross-linking density. Second, as the coating’s adhesion to the substrate relies on the bonding of acrylic polymers, a higher SA content proportionally reduces the concentration of emulsion molecules. This weakens the polymer’s ability to encapsulate the filler, thereby deteriorating the mechanical interlocking and leading to insufficient mechanical properties.

During substrate protection, the coating may be exposed to prolonged external mechanical forces such as mechanical friction, particle scouring, wear, and scratches. Therefore, excellent wear resistance can effectively mitigate the surface damage, maintaining the coating’s integrity and functionality. In this study, 600−mesh sandpaper was affixed under a 500 g load to abrade the coating surface for 50 and 100 cycles; the mass loss rate was recorded to evaluate wear resistance. As shown in Figure 5b, increasing the SA content from 0% to 18% led to a rising mass loss rate, which increased from 0.18% to 2.66% after 100 grinding cycles. At low cycle counts, the coating matrix was preferentially worn down under friction. As cycles increased, internal filler particles began to detach, triggering a “rapid failure stage” of wear. Notably, a higher SA content accelerated the mass loss rate, which was attributed to two mechanisms: (1) SA’s low mechanical strength makings its particles susceptible to interfacial debonding under sandpaper-induced shear forces, acting as wear weak points. Lu et al. found that the thermal conductivity of a coating gradually decreased to below 1 W/m·K after incorporating SA powder at a volume fraction of 20%. However, compared with the coating containing 5% SA, the wear resistance decreased from 219% to 99%. This phenomenon may be attributed to the presence of excessive hydrophobic SA powder: a large amount of SA created an increased interface area with the matrix, thereby reducing the cross-linking strength [34]. (2) The high SA loading diluted the matrix resin, reducing the latex encapsulation of the fillers and decreasing the interfacial bonding strength, thus promoting particle detachment during abrasion. Importantly, all samples exhibited low mass loss rates (<3%) after cyclic friction, confirming the SA coatings’ excellent wear resistance. Additionally, no surface scratches or cracks were observed.

### 2.5. Thermal Insulation 

**Figure 6:**
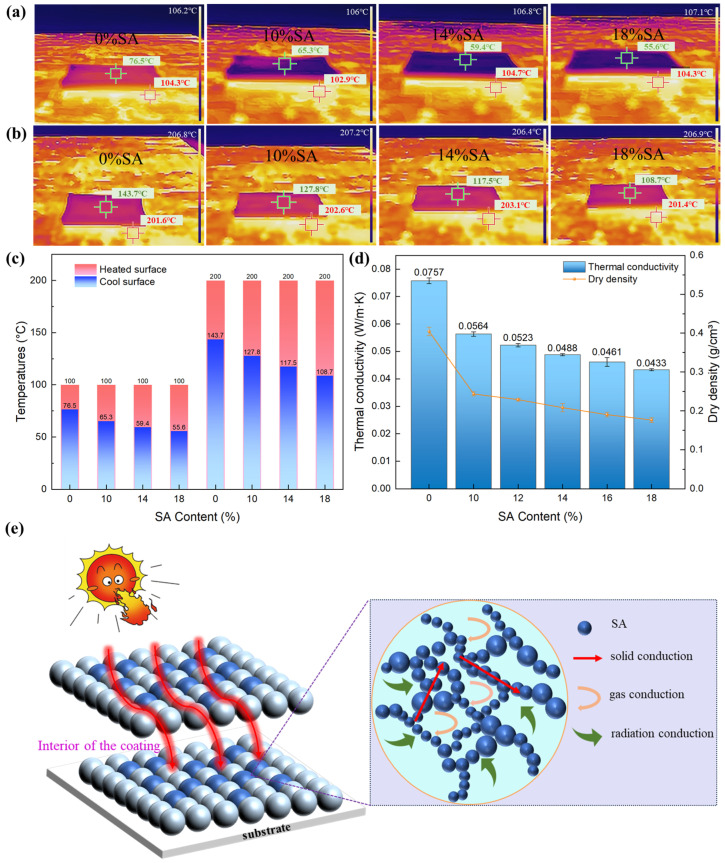
The thermal insulation effect of the coatings tested at different temperatures: (**a**) 100 °C and (**b**) 200 °C. (**c**) The thermal insulation temperature differences of samples. (**d**) The dry density and thermal conductivity characteristics of the coatings. (**e**) Internal thermal insulation mechanism diagram for the coating.

The thermal insulation coatings formulated with SA and HGMs demonstrated significant enhancements in both thermal insulation efficiency and lightweight characteristics. The Knudsen effect refers to a physical phenomenon in which gas−phase convective heat transfer is suppressed when pore sizes or structural features become comparable to the mean free path of gas molecules. In nanoscale confinement, the frequency of gas molecule collisions increases, disrupting the free movement of gas molecules and suppressing convective heat transfer [35]. This effect is especially significant with the low thermal conductivity of SA, which has a thermal conductivity of 0.023 W/m·K. The average pore size of SA is about 20–40 nm, while the average free path of air under standard conditions is about 69 nm [36]. In the nanoscale pore structure of SA, which is smaller than the mean free path of air molecules, the flow of gases within the nanopores is drastically constrained. The reduced space forces gas molecules to collide more frequently with the pore walls rather than with each other, effectively minimizing convective heat transfer. Given the ultra-small size of SA’s micropores, the contribution of internal gas convection to overall heat transfer is negligible. In addition to suppressing gas convection, SA’s intricate nanoporous network also impacts solid−phase heat conduction. The numerous nanopores fragment the direct heat conduction pathway, creating a tortuous “infinite path” effect. This significantly increases the effective length of heat transfer and reduces the efficiency of solid-phase heat conduction, further enhancing SA’s overall thermal insulation performance. He et al. investigated the influence of the amount of SA on the thermal conductivity of a polymer coating. The research showed that when the amount of SA was low, the thermal conductivity of the coating slightly decreased and most of the heat was still transferred through the polymer, which possessed low thermal resistance. When the content of SA was higher than 30%, the thermal conductivity of the coating rapidly decreased due to the higher amount of air in the pores, with the lowest thermal conductivity reaching below 0.1 W/m·K [37]. As shown in Figure 6d, with the increase in the amount of SA, the thermal conductivity and dry density showed a downward trend, indicating that the thermal insulation performance and lightweight application performance of the coating were enhanced. When the amount of SA increased to 18%, the thermal conductivity of the coating decreased from 0.0757 W/m·K to 0.0433 W/m·K (a decrease of 42.8%), and the density decreased from 0.404 g/cm^3^ to 0.177 g/cm^3^ (a decrease of 56.2%). The influence of heat transfer on the SA coating was affected by gas-solid coupled effect. However, the coatings with >18% SA suffered from high viscosity, poor fluidity, and workability.

Figure 6a,b respectively depict the surface temperature distribution of the coating when placed on a 100 °C and 200 °C hot plate. On the 100 °C hot plate, for the coating without SA, the cooling surface temperature rapidly increased to 76.5 °C within 10 min. For the coating with 18% SA, the cooling surface temperature increased to 55.6 °C at 10 min, which was 11 °C lower than that of the coating without SA. On the 200 °C hot plate, the upper surface temperature of the coating with 18% SA was 108.7 °C, exhibiting a temperature difference of 91.3 °C, which was 35 °C higher than that of the coating without SA (the upper surface temperature was 143.7 °C), indicating that the higher the temperature, the more obvious the difference in the thermal insulation effect was. Mechanistically, SA’s unique highly porous structure (low thermal conductivity) effectively impedes heat transfer, leading to a clear trend: a higher SA content correlates with a lower surface temperature and better insulation. Figure 6e summarizes the thermal insulation temperature differences for each sample, highlighting their ability to reduce heat loss and energy consumption.

### 2.6. Heat Resistance 

In terms of adhesion, organic coatings typically exhibit characteristics such as high hardness, exceptional waterproof performance, and robust bonding properties. Nonetheless, organic polymers are susceptible to thermal oxidation degradation at elevated temperatures, easily decomposing and releasing flammable gases when heated, leading to inferior heat resistance and consequently limiting their durability. At the same time, they are prone to softening, carbonization, or peeling at high temperatures, losing their protective function. As a result, organic coatings often face dual challenges in terms of flame retardancy and heat resistance [38]. Hence, it is of great significance to clarify the heat-resistant temperature of the coating. SA is an inorganic nanoporous material with exceptional thermal stability and low thermal conductivity. Its intricate porous network structure offers inherent fire resistance, effectively retarding flame propagation and absorbing combustion heat through the formation of a dense siliceous carbon layer during combustion [39]. The incorporation of SA is expected to enhance the heat resistance of organic coatings. A synchronized thermal analyzer was used to perform a thermal analysis and the gross calorific value (GCV) was measured.

**Figure 7:**
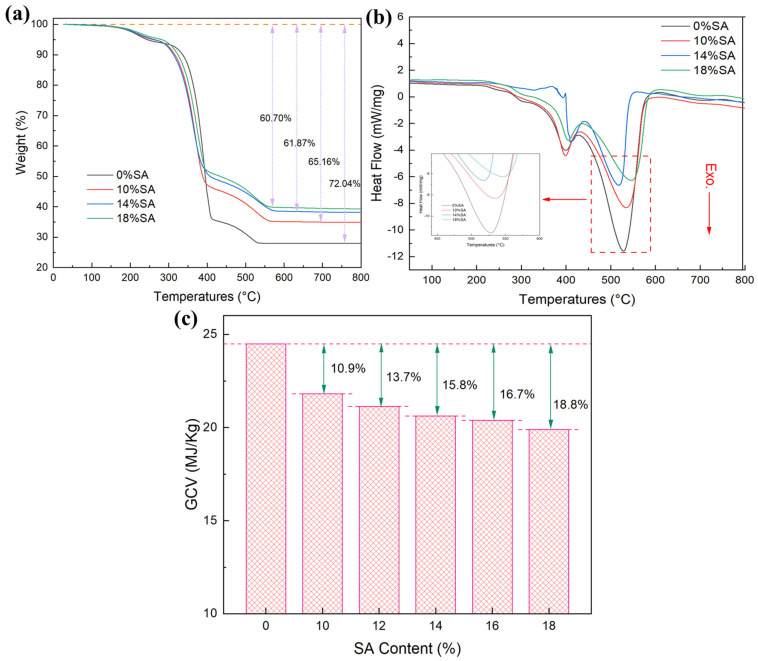
(**a**) TG, (**b**) DSC curves, and (**c**) GCV of coatings with different SA contents.

The TG-DSC curves of the coatings are illustrated in Figure 7. The TG analysis under an air atmosphere revealed that the 5% weight loss temperature of the coatings increased with SA content, with the 18% SA coating exhibiting the highest weight residue percentage of 39.3%, while the weight residue percentage of the coating without SA was 27.96% (Figure 7a). The observed trends in the residual rate of mass thermal decomposition underscore the efficacy of SA in fortifying the char residues of the coatings, confirming SA’s role in reducing weight loss and improving the heat resistance of the coatings through molecular chain entanglement with acrylic latex. Further analysis found that a large weight loss occurred at about 300–400 °C; this was mainly due to the thermal decomposition of acrylic organic molecules, and SA begins to exhibit weight loss at about 250−270 °C due to the thermal oxidation of Si-CH_3_ groups within the silica matrix [40,41]. Based on this, the main characteristics of the 120–400 °C stage were found to be due to the decomposition of the acrylic latex, SA, and additives. The second major weight loss occurred at about 400–550 °C, coinciding with the maximum heat release. Since HGMs’ inorganic glass phase is thermally stable and non-decomposable, and the residue of the SA-free coating exceeded the proportion of HGMs, the weight loss was inferred to have resulted from further decomposition of the residual organic latex. Above 550 °C, the weight residues stabilized as the organic latex completed thermal decomposition and remained as burning charcoal.

As the amount of SA increased, the mass proportion of the combustible organic matrix in the coating was diluted, resulting in a decrease in the total content of the combustible components per unit mass of the coating. The gross GCV of the coating without SA was 24.49 MJ/kg (Figure 7c). As the SA content increased, the GCV gradually decreased, and the minimum GCV was 19.89 MJ/kg. This reduction amounted to 18.8% compared to the coating without SA. This indicates that SA can reduce the GCV of organic polymer combustion through the physical dilution effect. This provides a reference for the optimization and improvement of the flame retardancy of these coatings.

## 3. Conclusions

In this study, coatings with thermal insulating effects were successfully prepared by joint mixing; hollow glass microspheres and SiO_2_ aerogel were integrated into an acrylic emulsion to develop lightweight thermal insulation coatings. The lowest thermal conductivity reached as low as 0.0433 W/m·K. Comprehensive testing demonstrated the coatings’ robust overall performance. Back-temperature measurements indicated that a 5 mm thick coating with SA maintained a cooling surface temperature of 108.7 °C when exposed to 200 °C, achieving a 91.3 °C thermal differential. This represents a 35 °C greater temperature gradient compared to the SA−free coating, confirming SA’s exceptional thermal insulation capability. Furthermore, the coatings demonstrated critical application-oriented properties including satisfactory abrasion resistance, corrosion resistance, and interfacial adhesion strength. Furthermore, the introduction of inorganic SA improved the heat resistance and reduced the GCV. However, the combustion heat value of the organic coatings was still relatively high. In future work, it is necessary to improve the flame retardancy to cope with fire hazards. It is noteworthy that excessive SA has an adverse effect on wear resistance, adhesion, and the water absorption rate. Thus, the balance among coating properties deserves comprehensive consideration. In addition, the energy saving efficiency of SA coatings for applications is worth investigating in follow-up work.

## 4. Materials and Methods

### 4.1. Raw Materials

Industrial-grade SA powders were purchased from Sinochem Hualu New Material Co., Ltd. (Chongqing, China). HGMs were purchased from Zhongke Huaxing New Materials Co., Ltd. (Taizhou, China). The acrylic emulsion (solid content was 47 ± 1%) and coupling agent KH550 (>99%) were purchased from Shandong Yousuo Chemical Co., Ltd. (Linyi, China). Defoamer 2299w (>99%) was purchased from Jiangxi Wuseshi New Materials Co., Ltd. (Jiujiang, China). The film-forming agent (>99%) was purchased from Juying Chemical New Materials Co., Ltd. (Shanghai, China). Polyether L62 (>99.5%) was procured from the Jiangsu Hai’an Petrochemical Plant (Haian, China). Cetyltrimethylammonium chloride (>99%) was purchased from Aladdin (Shanghai, China). The deionized water utilized in the experiments was prepared in the laboratory.

### 4.2. Preparation of Dispersed SA Slurry

The powder of lightweight SA is hydrophobic and difficult to directly disperse in waterborne coating systems. Instead, it tends to float and aggregate on the surface of the aqueous solution, failing to evenly disperse into the medium. Additionally, the nano-porous structure of SA, characterized by a high specific surface area and high surface energy, readily induces particle agglomeration. This uneven dispersion in the liquid medium compromises the structural uniformity and functional stability of the resulting coating. Therefore, in this study, the SA was introduced into the coatings in the form of a slurry. Polyether L62 and CTAC were dissolved in deionized water in a precise 1:4 ratio, and then SA with a mass fraction of 10 wt.% was added to the water dispersion. The mixture was thoroughly stirred at a frequency of 3000–5000 r/min in a high-speed shear disperser for about 10 min, and finally a fine cream-like SA slurry was obtained [42].

### 4.3. Preparation of Thermal Insulation Coatings

**Figure 8:**
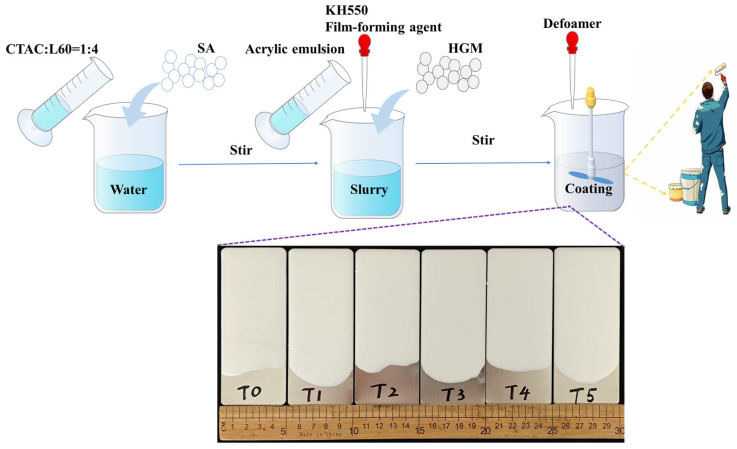
Schematic of the preparation process for the SA coatings.

For each quantity ratio, 10 g of acrylic emulsion was mixed with the pre-prepared SA slurry with different amounts of SA (0%, 10%, 12%, 14%, 16%, and 18%) for 5–10 min (500–600 r/min). After that, 2 g of HGMs was added, and the mixture was stirred at 800–1000 r/min for about 10 min. During this process, 1% KH550, 4% film-forming agent, and 1% defoamer were added sequentially with stirring (Figure 8). The resulting coating was applied to pre-polished and cleaned tinplate sheets (d = 0.28 mm), which were then cured at 45 °C for 48 h to achieve full cross-linking. The dry coating thickness was measured to be 2.0 ± 0.3 mm. The SA-free sample was designated T0, while the other five samples containing 10%, 12%, 14%, 16%, and 18% SA slurry were denoted as T1, T2, T3, T4, and T5, respectively.

### 4.4. Characterization Methods

The thermal conductivity of the coating was measured with a thermal conductivity meter (TC3000E, XIATECH, Xi’an, China) following the transient hot wire method. The dry density of the sample was obtained by measuring the mass and volume of the shaped regular sample and using the density calculation formula. The microstructure and surface morphology of the samples were observed using a field emission scanning electron microscope (Sigma 300, Zeiss, Oberkochen, Germany) at an accelerating voltage of 3 kV for morphological imaging. To enhance the resolution for SEM observation, the composite specimens were gold−sputtered. According to HG/T 3344-2012 “Determination Method of Water Absorption Rate of Paint Films” [29], the coating was applied to a 120 × 25 × 0.2–0.3 mm tinplate sheet. After drying, the test plate was vertically immersed in water. After 24 h, the test plate was taken out, and the surface moisture of the coating was quickly absorbed with filter paper and immediately weighed. The water absorption rate W of the coating was expressed as a percentage, and the formula was as follows:

$$w=\frac{m_{2}-m_{1}}{m_{1}-m_{0}}$$

where $m_{0}$ represents the weight of the base plate, $m_{1}$ represents the weight of the test plate before immersion in water, and $m_{2}$ represents the weight of the test plate after immersion in water.

To assess the hydrophobicity of the coatings, the water drop method was utilized. Specifically, contact angles after depositing 2 μL of deionized water onto the sample surface were measured using an automatic contact angle meter (model ASR-705S, Guangdong Aisry Instrument Technology Co., Ltd., Dongguan, China).

Adhesion tests were conducted according to GB/T 9286–2021 “Paints and varnishes–cross-cut test” (China, 2021) [43]. The grid cutter was held perpendicularly to the surface of the sample and a uniform force was applied to cut through the coating to the surface of the substrate, creating two sets of mutually perpendicular parallel lines to form a 5 × 5 grid array at specific intervals on the surface of the coating. The degree of coating peeling was assessed using tape. Meanwhile, following GB/T 5210-2006 “Paints and varnishes−Pull off test for adhesion” (China, 2006) [44], the metal test column was adhered to the coating surface with adhesive and a cutting tool was used to cut along the edge of the test column to the substrate. It was stretched vertically at a constant rate to measure the maximum tensile force when the coating peeled off.

To test the corrosion resistance of the coating across diverse environments, the coating was submerged in 3.5 wt.% NaCl and 5 wt.% NaOH solutions, and observed after 7 days. The wear resistance was measured using the weight loss wear test: 600−mesh sandpaper was combined with a load pressure of 500 g to polish the whole surface of the coating on the substrate for a specific number of cycles. The wear resistance was calculated as follows:

$$C=\frac{m_{0}-m_{1}}{m_{0}}$$

where $m_{0}$ represents the weight of the coating, $m_{1}$ represents the weight of the coating after the wear test, and C is the mass loss rate.

To analyze the thermal insulation performance, the samples were evaluated by measuring the temperature difference across the heated coating. Heating plates set at 100 °C and 200 °C were used as the heat sources. Once the heat source temperature stabilized, 5 mm thick coatings with varying SA contents were placed flat on the heating plates, and their surface temperatures were recorded for 10 min using an infrared thermal imager. The thermal stability analysis was performed using TG–DSC (STA8000-FTIR-GCMS-ATD, Shanghai, China) with a heating rate of 10 °C/min from room temperature to 800 °C in an air atmosphere. The GCV of the SA composite coatings was measured using an oxygen bomb calorimeter.

## Data Availability

The original contributions presented in this study are included in the article. Further inquiries can be directed to the corresponding authors.
